# Hope for success as a mediator between Big Five personality traits and achievement goal orientation among high performance and recreational athletes

**DOI:** 10.1371/journal.pone.0288859

**Published:** 2024-03-21

**Authors:** Maciej Tomczak, Paweł Kleka, Ewa Tomczak-Łukaszewska, Małgorzata Walczak

**Affiliations:** 1 Department of Psychology, Poznan University of Physical Education, Poznań, Poland; 2 Faculty of Psychology and Cognitive Sciences, Adam Mickiewicz University in Poznan, Poznań, Poland; 3 Faculty of English, Adam Mickiewicz University in Poznan, Poznań, Poland; Julius-Maximilians-Universität Würzburg, GERMANY

## Abstract

The main objective of this study was to determine whether hope for success mediates the relationship between personality and goal orientation in high performance and recreational athletes. The cross-sectional study included 289 high performance and recreational athletes of various sports (age: M = 20.34, SD = 1.86). To examine personality, we used the Big Five IPIP-BFM-20 questionnaire. To assess hope for success, we used the Hope for Success Questionnaire. The Task and Ego Orientation in Sport Questionnaire (TEOSQ) was employed to examine goal orientation. Hope for success significantly mediates the relationships between conscientiousness, extraversion, emotional stability, intellect, and task goal orientation in sport. Higher levels of these personality traits are related to athletes’ higher hope for success, which, in turn, is positively related to their task orientation. Personality traits may underpin the hope for success that develops from childhood. This, in turn, appears to be an important component on which, combined with the positive interaction and support from coaches, a task goal orientation beneficial to sport can be shaped in athletes.

## Introduction

The construct of goal orientation holds a very prominent spot in the field of research on motivational processes in sport. This is largely a consequence of research results that have repeatedly highlighted the importance of goal orientation for functioning in sport [1, 2]. While there are different approaches to goal orientation, its most basic classification–that found in Achievement Goal Theory [3–6]–consists of two components, i.e. *task orientation* related to one’s orientation towards development, learning and evaluating one’s own skills independently of performance of others, and *ego orientation*, where individuals evaluate their skills in comparison to other people [3, 4, 7–9]. Both task orientation and ego orientation are frequently examined to uncover their potential conditionings, including personality traits. Little research, however, has addressed the importance of personality traits in the development of a goal orientation favourable in sport. In our view, a rather important role in this context, and thus in the relationship between personality and goal orientation, may be played by *hope for success*.

One of the most well-known definitions of hope is Snyder’s [10–14] construct. According to Snyder, hope pertains to an individual’s beliefs about achieving a goal. Hope enables a person to initiate the pursuit of a set goal and to persist in achieving it despite setbacks and obstacles. In this sense, hope concerns an individual opinion of having ‘strong willpower’ (*agency*). Moreover, hope is also related to self-beliefs about one’s resourcefulness and ability to provide solutions (*pathways*), e.g. in difficult situations. These beliefs become activated as thoughts and accompany goal-oriented activities [10–17]. Snyder’s concept of hope concerns hope for success as it refers to one’s belief that one will succeed and that this success will be the result of one’s own competences [15]. Earlier studies have shown that hope is important for effective functioning in sport in terms of a positive relationship with athletic performance [18, 19], it prevents professional burnout among athletes [20], and it promotes athletes’ well-being and their rehabilitation after sports injuries [21].

Personality correlates of goal orientation/achievement goals and hope have been studied using the Big Five Model by Costa and McCrae [22, 23]. It comprises neuroticism related to lower emotional stability, extraversion related to sociability and activity, openness (intellect) linked to preference for new stimuli and situations, agreeableness connected to cooperative attitude, and conscientiousness, whose correlates are perseverance and being methodical [24]. The importance of these characteristics in the interaction with environmental factors (parents’ and guardians’ activity, support from the coach, etc.) can be important for the formation of a certain goal orientation in sport. It has been found, for example, that low neuroticism, high conscientiousness, extraversion, openness and agreeableness are related to more favourable forms of goal orientation linked to an orientation towards learning new skills and mastery [25]. The role of a favourable family and educational environment is also often highlighted when considering the determinants of the development of hope, as beliefs about hope are already shaped from childhood [15]. Alongside these factors, however, emphasis can also be placed on the impact of personality traits acting as a specific developmental context interacting with the mentioned factors. Although there are some differences in the results of the studies [26–30], on the whole it can be stated that conscientiousness, emotional stability, extraversion, openness, and agreeableness are positively related with hope.

Despite the mentioned relations of personality traits with achievement goal orientation, however, to the best of our knowledge there are hardly any studies addressing the potential explanation of this relationship in sport. Exploring the mechanism of these relationships can promote an understanding of the possibility of forming a beneficial goal orientation in athletes that depends in part also on their personal conditionings shaped from an early developmental stage. In our opinion, hope for success in athletes may have an important role in understanding the mentioned relations between personality and goal orientation in sport. Therefore, the main aim of our work is to determine the mediating role of hope for success in the relationship between personality traits and goal orientation in sport. We assume that hope for success, on the one hand, depends partially on an individual’s personality and the influences from their guardians, and, on the other hand, increases the chance of a goal orientation favourable in sport. Our study is cross-sectional in its nature, hence the direction of the hypothesized relationships will emerge from the substantive assumptions and previous research. Cross-sectional studies are unfortunately not free from shortcomings such as limited cause-effect inference [31]. However, our study can serve as a good starting point for further more advanced research, e.g. longitudinal studies, which can further verify the relationships that we have obtained. Despite the data indicating some potential for personality change [32, 33] induced i.a. by activity related to certain motivational tendencies [34], what is often assumed is the importance of personality traits for the development of goal orientation [35] and what is evaluated is the predictive value of personality traits for goal orientation [36, 37]. As indicated earlier–analogous to that of goal orientation–there are also studies of the personality conditionings of hope [26, 38]. It is also worth noting that in our study, only goal orientation relates to sport (the scale: Task and Ego Orientation in Sport Questionnaire), while personality traits and hope for success are of general nature. We can assume here that being relatively genetically determined, personality traits seem to serve as a baseline here. Then, it is in interaction with them that hope for success develops from childhood onwards. Once children or adolescents enter sports environment, a sports goal orientation begins to form. Despite the important role of the motivational climate in sport in developing goal orientation [39, 40], young individuals–at the beginning of their engagement in sport–already possess certain personality dispositions and general beliefs about hope for success. Those beliefs may be more or less conducive to the shaping of a particular goal orientation in the sports environment.

Following previous research results, the assumption can be made that individuals with a higher level of conscientiousness are more persistent and determined in the pursuit of their goals and thus may achieve their goals more effectively. As a result of this, they may experience the sense of success and success-driven positive emotions more often, develop positive thoughts about their goal attainment and about themselves [41], and they may receive more favourable feedback from their caregivers and teachers, which consequently contributes to their developing a higher hope for success. In turn, the generalized beliefs about coping with different situations and willpower (i.e. hope in Snyder’s [10] view), both formed since childhood, may foster a more task-oriented goal orientation when at some point in their lives people become immersed in a sport context. Task orientation is after all tied to goal achievement through high levels of effort and involvement [8], which are also conceptually linked to a high level of hope [12]. Thus, it can be assumed that for people with high levels of hope, a focus on task is natural and it will be easier for them to form a task orientation in sport. These positive correlations between learning (task) orientation and hope were reported in earlier studies [42]. Likewise, we hypothesize that high levels of extraversion related to activity and experiencing positive emotions may foster higher effectiveness and, consequently, higher hope for success, which increases the chances of being highly task-oriented in sport. In turn, lower emotional stability (neuroticism), may be combined with lower emotional resilience related to less effective coping with stressors and more frequent experience of negative emotions during task performance / goal achievement, which ultimately–in line with the previously presented results–does not favour hope for success and subsequently fails to promote task orientation in sport as well. It is also possible that openness (intellect), related to creativity, and agreeableness, related to a cooperative attitude, also foster a greater hope for success [26–28] and ultimately task orientation. It is also likely that hope for success mediates the relationship between personality and ego orientation associated with an orientation towards winning in sport, although this issue remains inconclusive. Earlier studies involving university students have shown a significant weak but positive relationship between the component of hope (pathways) and performance (ego) orientation [42]. Perhaps, then, one’s beliefs about having the ability to cope with a difficult situation (hope for success) may promote one’s effectiveness also in ego-orientation-based sports rivalry. This issue will also be addressed in the present study.

We also assume that the analyzed mediational relationships may differ between high performance and recreational athletes. Prior research, for example, found recreational and high performance athletes to differ in terms of their level of psychological traits, their motives and outcome expectations [43–45]. In high performance sport, inherently driven by the focus on maximizing performance in a challenging competitive situation, personality variables may carry different weight for functioning than in recreational sport. For this reason, we assume that the type of sport (high performance/recreational) will act as a moderating variable.

## Materials and methods

### Participants

The study was carried out from April 2022 to July 2022 on 289 participants (their age: *M* = 20.34, *SD* = 1.86). In the group, there were 165 high performance athletes who regularly competed in sport tournaments and 124 recreational athletes who participated in sports activities that involved competition and sometimes took part in tournaments (100 females, 189 males). The subjects of the study were obtained through convenience sampling from various faculties related to sport, physical activity and health from the University of Physical Education in Poznań, a typical university running faculties of this type in a large city of Poland. All high performance athletes declared that they practiced sport with the main aim of achieving maximum sport achievement. They often trained in sports clubs not affiliated with the university. Recreational athletes declared that they trained mainly for health and improving physical fitness. The sport disciplines that were most often trained by the athletes in the study were football, volleyball, running, basketball, dance, and swimming. The study was anonymous. The description of the research was reviewed by the Bioethics Committee at Poznan University of Medical Sciences (Poland). The Committee issued a statement that the research did not bear the features of a medical experiment and in accordance with the Polish law and GCP was not subject to the opinion of the Bioethics Committee (Statement No. KB -273/22). Following recommendations of the Committee, the study instructions informed the participants that taking part in the study and submitting the completed questionnaire would be considered as their informed consent to take part in the present study. The study was conducted in line with the Declaration of Helsinki. All methods were carried out in compliance with relevant guidelines and regulations. The study was conducted in the frame of a larger research project investigating measurement and psychosocial correlates of goal orientation in high performance and recreational athletes regarding physical activity they undertake.

### Research tools

A short questionnaire IPIP-BFM-20 [46] from the Big Five personality model was used to measure personality traits. The questionnaire derives from the lexical model of Goldberg’s [47, 48] and is used to determine the levels of five traits, i.e. extraversion, agreeableness, conscientiousness, emotional stability, and intellect. The questionnaire consists of 20 statements (with four statements for each trait). A respondent’s task is to evaluate how well the statement applies to them using a scale from one to five [46]. The validity of the questionnaire was assessed using–among other ways–factor analysis and correlations of the questionnaire with other instruments aimed at measuring personality from the Big Five perspective. As reported by the authors [46], the obtained correlations between the personality traits measured with the IPIP-BFM-20 and the corresponding personality traits assessed with NEO-PI-R, IPIP-NEO-PI-R, BFAS, and IPIP-45AB5C scales confirm the validity of the IPIP-BFM-20 questionnaire. What is more, personality traits of the short version correlated strongly with those of the long version of the IPIP-BFM-50. The differences in the correlation coefficients of the two versions (i.e. short and long) with other scales were small and did not exceed a value of 0.12. Reliability as determined by Cronbach’s alpha was tested in two groups and ranged from 0.65 to 0.78 [46]. As pointed out by Donnellan et al. [49], the longer-term (i.e. up to 9 months) test-retest correlations for the short version of the IPIP scales (Mini-IPIP scales) were: extraversion (*r* = 0.86), agreeableness (*r* = 0.68), conscientiousness (*r* = 0.77), neuroticism (*r* = 0.82), and intellect (*r* = 0.75). The authors report that these coefficients resemble the values obtained for the longer version of the IPIP-BFM scales [49].

The Polish version of Snyder’s scale [10], in the Polish adaptation of Łaguna et al. [15] (known as The Hope for Success Questionnaire), was used in the study. The Hope for Success Questionnaire consists of 12 test items. It is used to determine the level of hope for success and its two components, i.e. problem-solution skills (*pathways*) and willpower (*agency*). The respondents’ task is to assess how accurately each statement applies to them on a scale ranging from one to eight. Eight test items (four for each component) are used in the calculations. Since Snyder’s construct of hope takes into account an individual’s beliefs that they will succeed and this success results from their own competence, the authors of the Polish adaptation named the Polish version of Snyder’s Hope Scale as the Hope for Success Questionnaire [15]. The validity of the questionnaire was evaluated with factor analysis and correlations of the questionnaire with other tools. The analysis revealed correlations with conceptually similar constructs such as, for example, self-efficacy (Generalized Self-Efficacy Scale–GSES), basic hope (Basic Hope Questionnaire–BHI), optimism (Revised Life Orientation Test–LOT-R), and self-esteem (The Rosenberg Self-Esteem Scale–SES). As part of the validity testing, it was also shown that people with higher levels of hope for success deal with difficult situations more effectively, e.g. they are more committed to reconstructing their houses after the flood disaster or to seeking a bank loan, and they are more prone to taking measures on their own to find their way out of unemployment. The average Cronbach’s alpha coefficient of reliability obtained across a range of studies was 0.82. The test-rest reliability, determined with a two-month interval, was 0.83 [15].

The Task and Ego Orientation in Sport Questionnaire (TEOSQ) [7, 8] in the Polish adaptation of Tomczak et al. [50] was employed in the present study. The questionnaire is made up of 13 test items. Seven of the items address task orientation and six target ego orientation. The respondents’ task is to indicate how well each statement describes them using a scale from one to five. The validity of the TEOSQ was determined by means of factor analysis and by examining the correlations of the TEOSQ with the Sport Motivation Scale (SMS) and the respective differences in the groups distinguished on the basis of content. As expected, there were high correlations between the task subscale and the SMS dimensions of intrinsic motivation, as well as a relationship between the ego subscale and external regulation. It was also found that individual sport athletes are more ego-oriented and less task-oriented than team sport athletes. These effects are particularly pronounced in high performance athletes. Reliability, as determined by Cronbach’s alpha coefficients, was 0.84 for the ego subscale and 0.81 for the task subscale. The test-rest reliability, assessed with a two-week interval, was 0.86 for both subscales [50].

### Statistical analysis

In the first step, we carried out an analysis of descriptive statistics and examined indicators related to the shape of the distribution: skewness and kurtosis. Next, we computed r-Pearson correlations between the investigated variables in order to check the relationship and convergence between the examined constructs [51].

A moderated mediation analysis was performed to verify the assumption that hope for success is a mediator in the relationship between personality traits and goal orientation of high performance and recreational athletes. Personality traits were set as independent variables, hope for success as the mediator, whereas the dependent variable was task goal orientation, and then ego goal orientation. Further, it was tested whether the type of sport activity (i.e. high performance/recreational) moderates the mediation effects. Following the current recommendations, to verify the mediation effect, we analyzed the significance of the indirect effect [using the bootstrap method, we analyzed the significance of the product of the path coefficients between the independent variable and the mediator (path a) and the mediator and the dependent variable (path b)]. Using this approach, to obtain a significant mediation, it is not necessary to obtain a significant primary relationship between the independent variable and the dependent variable (total effect), which was the case in the earlier mediation analysis approach of Baron and Kenny (the causal steps approach). We have therefore assumed, in line with current recommendations, that for a mediation effect to be obtained, a statistically significant indirect effect is required [52, 53]. The percentile bootstrap was applied to test for significance of indirect effects, moderating effects, and direct effects, based on 10.000 bootstrap samples. The 95% confidence intervals are reported. It is assumed that an effect is statistically significant when the confidence interval does not contain a zero value [52, 53]. It is often assumed that the minimum sample size in regression analysis should be 15 observations per predictor in the model. Hence, we assume that the sample size obtained by us, will be sufficient. The statistical analyses were carried out using Jamovi software (ver. 2.3.21) and data are available in S1 File.

In cross-sectional studies, the choice of direction of the relationship between variables is justified theoretically and can often be considered quite conventional. In such cases, a further analysis can be carried out to strengthen the justification for the choice of direction of the relationship between variables and thus the choice of a mediator. As pointed out in the *Introduction* of the present article, in our model personality traits–being biologically-based and most basic in nature–are the independent variables. We assumed that they are of primary importance for the development of hope for success and goal orientation. Accordingly, our mediation model assumed the following relationships: *personality traits–hope for success–goal orientation in sport*. However, owing to the cross-sectional nature of the study, a different direction of the relationship could also be assumed, notably between hope for success and goal orientation. We could try to make an assumption here that personality traits increase the chance of a high goal orientation in sport, which, in turn, increases the chance of a high level of hope for success (the assumed direction of the relationship: *personality traits–goal orientation in sport–hope for success*). In such a case, consistent with the analysis presented by Hayes [52], in order to substantiate the mediator status and support empirically which variable (hope for success or goal orientation) fits better as a mediator, we conducted further analyses. In these, the relationship between the variables was reversed, i.e. goal orientation in sport was introduced as a mediator, and hope for success was set as the dependent variable. The mediation relation obtained in each case, however, was weaker relative to the mediation relation assumed in the *Introduction* of this article: where hope for success is a mediator (in each case, smaller standardized effect sizes were obtained). Further, with goal orientation as a mediator, a mere two out of five mediation relations were significant, whereas with hope for success as a mediator, four out of five mediation relations reached significance. This analysis provides more empirical justification for choosing hope for success as a mediator and its summary was presented in the last paragraph of the *Results* section.

### Common method variance

Obtaining information in questionnaires from a single person involves the risk of Common Method Variance (CMV) problem. A Harman single-factor-analysis (SFA) was conducted to verify this issue [54]. Following the guidelines of this analysis, it was examined how much of the variance is explained by a single factor when all detailed items from the questionnaires are included. It is assumed that if a single factor explains more than 50% of the variance, then there is a Common Method Variance (CMV) issue. In our case, when all items from the questionnaires were included on one factor, the single factor explained 15.42% of the variance. This value is less than 50%. In addition, at least one researcher participated personally in each study, who, among other things, assured the participants of the full anonymity of the study and of the confidentiality of the results by asking for careful reflection on each item. He also ensured that the study is voluntary and that any participant can withdraw at any time. Furthermore, standardized and recognized psychological questionnaires were used in the study, where the selection of items was carefully considered.

## Results

Table 1 shows the descriptive statistics for the investigated variables. In each case, the values of skewness and kurtosis did not exceed the absolute value of 1.

**Table 1:** Descriptive statistics for the investigated variables.

Characteristics	Mean (SD)	Skew	Kurtosis	SE
E	3.53 (0.97)	-0.68	-0.08	0.06
A	3.72 (0.73)	-0.58	0.76	0.04
C	3.31 (0.88)	-0.44	-0.38	0.05
ES	2.91 (0.90)	-0.04	-0.65	0.05
I	3.71 (0.71)	-0.50	0.54	0.04
HS	48.56 (7.05)	-0.33	0.56	0.41
TASK	31.07 (3.37)	-0.79	0.10	0.20
EGO	20.06 (4.76)	-0.36	0.08	0.28

E–extraversion, A–agreeableness, C–conscientiousness, ES–emotional stability, I–intellect, HS–hope for success, TASK–task orientation, EGO–ego orientation.

Significant positive relationships were found between extraversion, conscientiousness, emotional stability, intellect, and hope for success. What is more, hope for success, conscientiousness and intellect were positively related with task orientation, and intellect was also related with ego orientation (Table 2).

**Table 2:** Correlations between the investigated variables.

Correlation Matrix	E	A	C	ES	I	HS	TASK
HS	0.31 ***	0.11	0.24 ***	0.33 ***	0.57 ***	—	—
TASK	0.06	0.11	0.17 **	0.05	0.20 ***	0.33 ***	—
EGO	0.02	-0.05	0.03	0.04	0.14 *	0.09	0.09

Note.

* p < 0.05

** p < 0.01

*** p < 0.001.

E–extraversion, A–agreeableness, C–conscientiousness, ES–emotional stability, I–intellect, HS–hope for success, TASK–task orientation, EGO–ego orientation.

**Fig 1:**
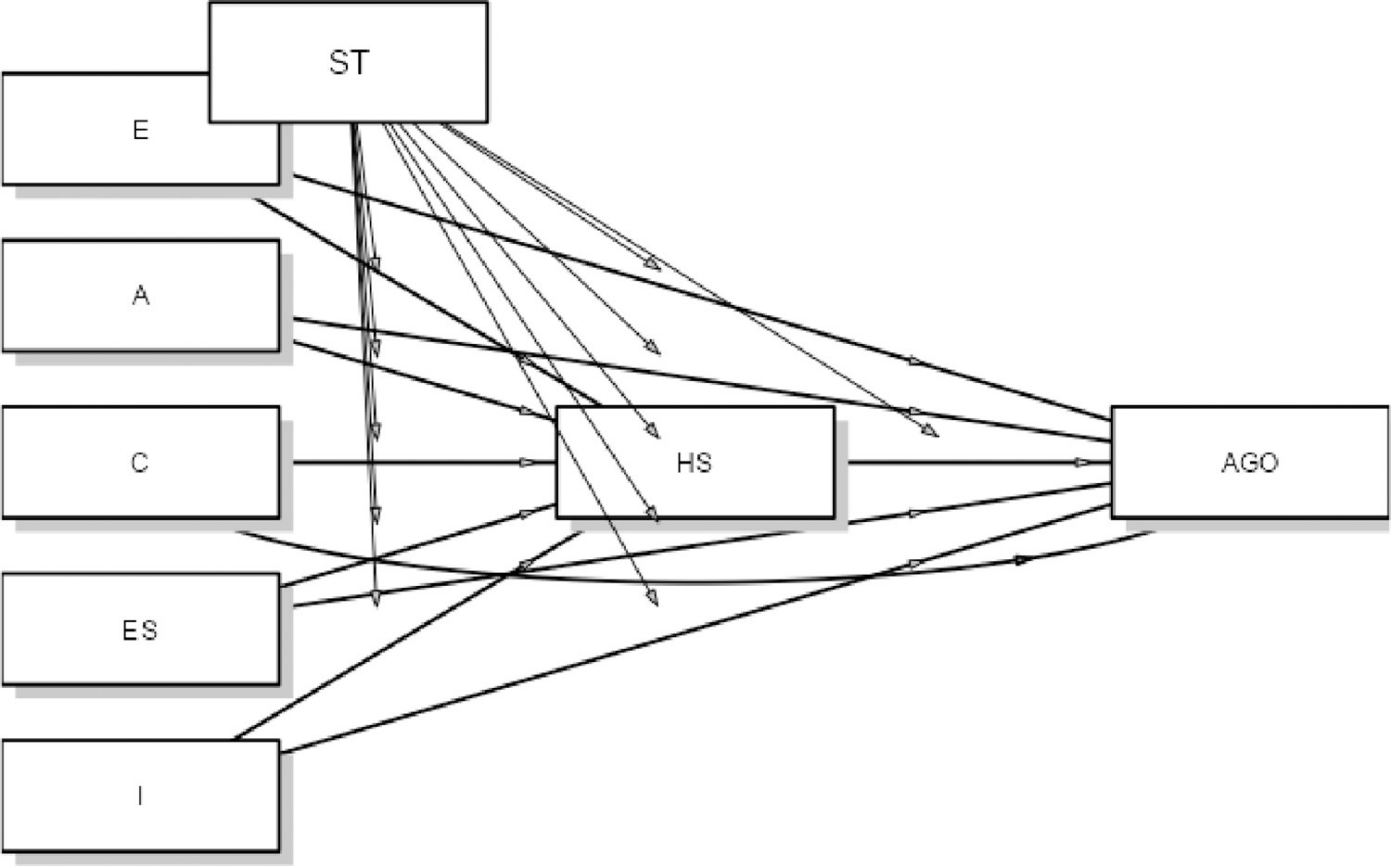
Hope for success as a mediator in the relationship between personality traits and achievement goal orientation in high performance and recreational athletes–a moderated mediation model.

Fig 1 shows an estimated moderated mediation model where the independent variables are personality traits, the mediator is hope for success and the dependent variable is the athletes’ goal orientation. The moderator is the type of undertaken sport activity i.e. high performance/recreational. Two models were estimated: in the first model the dependent variable was athletes’ task orientation and in the second model the dependent variable was their ego orientation.

### Moderated mediation analysis for task orientation–hope for success as a mediator in the relationship between personality traits and achievement task orientation of athletes

Table 3 shows the interaction (moderation) effects for all pathways of the mediation model for task orientation of athletes.

**Table 3:** Interaction (moderation) effects for all pathways of the mediation model for task orientation of athletes.

Interaction	Estimate	SE	95%CI Lower	95% CI Upper	β
E:ST ⇒ HS	-0.7431	0.7630	-2.2285	0.7659	-0.0513
A:ST ⇒ HS	0.2933	0.9729	-1.7072	2.1017	0.0153
C:ST ⇒ HS	-0.6288	0.7760	-2.0624	0.9788	-0.0389
ES:ST ⇒ HS	1.1649	0.7752	-0.4393	2.5829	0.0744
I:ST ⇒ HS	-1.1500	1.0276	-3.1403	0.8808	-0.0581
E:ST ⇒ TASK	-0.4684	0.4241	-1.3143	0.3486	-0.0673
A:ST ⇒ TASK	0.8493	0.5467	-0.2312	1.9055	0.0921
C:ST ⇒ TASK	0.1600	0.4948	-0.8396	1.1177	0.0206
ES:ST ⇒ TASK	0.0598	0.4502	-0.7942	0.9847	0.0080
I:ST ⇒ TASK	0.5619	0.7457	-0.8502	2.0588	0.0591
ST:HS ⇒ TASK	-0.0688	0.0741	-0.2151	0.0743	-0.4932

ST–sport type (high performance/recreational), HS–hope for success, E–extraversion, A–agreeableness, C–conscientiousness, ES–emotional stability, I–intellect, TASK–task orientation.

For none of the tested relationships between variables did the interaction effect reach statistical significance (in each case the confidence intervals contained a zero value) (Table 3). Hence, the sport type did not turn out to be a significant moderator in the relationships between the variables. It can thus be assumed that the relationships between the variables were comparable in high performance and recreational athletes. Therefore, the combined average mediation effects are presented with no breakdown into high performance and recreational athletes. The results of the mediation analysis for task orientation of athletes are presented in Table 4.

**Table 4:** Mediation effects for task orientation of athletes.

Effect	Type	Estimate	SE	95%CI Lower	95% CI Upper	β
E ⇒ HS ⇒ TASK	Indirect	0.1447	0.0711	0.0248	0.3039	0.0416
A ⇒ HS ⇒ TASK		-0.0134	0.0763	-0.1626	0.1452	-0.0029
C ⇒ HS ⇒ TASK		0.2449	0.0819	0.1014	0.4187	0.0635
ES ⇒ HS ⇒ TASK		0.1974	0.0737	0.0736	0.3661	0.0527
I ⇒ HS ⇒ TASK		0.7424	0.1917	0.3927	1.1601	0.1564
E ⇒ HS	Component	0.9419	0.3807	0.1809	1.6695	0.1299
HS ⇒ TASK		0.1536	0.0362	0.0845	0.2287	0.3200
A ⇒ HS		-0.0869	0.4832	-1.0073	0.8847	-0.0091
C ⇒ HS		1.5946	0.3866	0.8121	2.3373	0.1983
ES ⇒ HS		1.2853	0.3917	0.5452	2.0651	0.1648
I ⇒ HS		4.8340	0.5136	3.8147	5.8655	0.4886
E ⇒ TASK	Direct	-0.1682	0.2157	-0.5929	0.2559	-0.0484
A ⇒ TASK		0.3809	0.2725	-0.1391	0.9245	0.0827
C ⇒ TASK		0.3579	0.2464	-0.1160	0.8510	0.0927
ES ⇒ TASK		-0.1792	0.2253	-0.6391	0.2329	-0.0479
I ⇒ TASK		0.1075	0.3714	-0.5928	0.8369	0.0226
E ⇒ TASK	Total	-0.0108	0.2176	-0.4283	0.4268	-0.0031
A ⇒ TASK		0.3625	0.2794	-0.1641	0.9329	0.0790
C ⇒ TASK		0.6136	0.2465	0.1305	1.0867	0.1595
ES ⇒ TASK		-0.0018	0.2319	-0.4695	0.4298	-0.0005
I ⇒ TASK		0.8697	0.3172	0.2928	1.5321	0.1838

HS–hope for success, E–extraversion, A–agreeableness, C–conscientiousness, ES–emotional stability, I–intellect, TASK–task orientation, β–standardized value.

Statistically significant indirect effects were obtained for extraversion, conscientiousness, emotional stability, and intellect. Hope for success was thus found to be a mediator between these traits and task orientation of athletes. Statistically significant positive relationships were obtained between extraversion, conscientiousness, emotional stability, intellect, and hope for success. A positive relationship was also found between hope for success and task orientation of athletes (Table 4). A moderated mediation analysis was then conducted for athletes’ ego orientation.

### Moderated mediation analysis for ego orientation–hope for success as a mediator in the relationship between personality traits and achievement ego orientation of athletes

Table 5 shows the interaction (moderation) effects for all pathways of the mediation model for ego orientation of athletes.

**Table 5:** Interaction (moderation) effects for all pathways of the mediation model for ego orientation of athletes.

Interaction	Estimate	SE	95%CI Lower	95% CI Upper	β
E:ST ⇒ HS	-0.7430	0.7553	-2.2209	0.7297	-0.0512
A:ST ⇒ HS	0.2933	0.9816	-1.7289	2.1619	0.0153
C:ST ⇒ HS	-0.6288	0.7764	-2.0902	0.9732	-0.0389
ES:ST ⇒ HS	1.1648	0.7787	-0.4226	2.6037	0.0744
I:ST ⇒ HS	-1.1501	1.0226	-3.1445	0.8622	-0.0581
E:ST ⇒ EGO	0.1189	0.6526	-1.2216	1.3505	0.0121
A:ST ⇒ EGO	0.4806	0.9377	-1.3615	2.3253	0.0370
C:ST ⇒ EGO	0.2458	0.7025	-1.1748	1.5888	0.0225
ES:ST ⇒ EGO	-0.8286	0.7641	-2.2878	0.7145	-0.0784
I:ST ⇒ EGO	0.0869	1.2062	-2.2349	2.5500	0.0065
ST:HS ⇒ EGO	0.0342	0.1165	-0.1957	0.2590	0.1743

ST–sport type (high performance/recreational), HS–hope for success, E–extraversion, A–agreeableness, C–conscientiousness, ES–emotional stability, I–intellect, EGO–ego orientation

As in the case of task orientation, for none of the relationships between variables did the interaction effect reach statistical significance (in each case the confidence intervals contained a zero value) (Table 5). It can therefore be assumed that the relationships between the variables were similar in high performance and recreational athletes. The combined average mediation effects are therefore presented without splitting high performance and recreational athletes. The results of the mediation analysis for ego orientation of athletes are presented in Table 6.

**Table 6:** Mediation effects for ego orientation of athletes.

Effect	Type	Estimate	SE	95%CI Lower	95% CI Upper	β
E ⇒ HS ⇒ EGO	Indirect	-0.0041	0.0598	-0.1328	0.1146	-0.0008
A ⇒ HS ⇒ EGO		0.0004	0.0282	-0.0659	0.0574	0.0001
C ⇒ HS ⇒ EGO		-0.0069	0.0937	-0.1926	0.1808	-0.0013
ES ⇒ HS ⇒ EGO		-0.0055	0.0793	-0.1676	0.1535	-0.0011
I ⇒ HS ⇒ EGO		-0.0208	0.2810	-0.5565	0.5557	-0.0031
E ⇒ HS	Component	0.9419	0.3759	0.2140	1.6741	0.1299
HS ⇒ EGO		-0.0043	0.0582	-0.1200	0.1098	-0.0064
A ⇒ HS		-0.0869	0.4865	-1.0090	0.9153	-0.0091
C ⇒ HS		1.5946	0.3896	0.8106	2.3328	0.1983
ES ⇒ HS		1.2853	0.3888	0.5356	2.0749	0.1648
I ⇒ HS		4.8340	0.5073	3.8403	5.8525	0.4886
E ⇒ EGO	Direct	-0.0267	0.3286	-0.6475	0.6356	-0.0055
A ⇒ EGO		-0.4339	0.4642	-1.3204	0.5133	-0.0669
C ⇒ EGO		0.1082	0.3535	-0.5648	0.8284	0.0199
ES ⇒ EGO		0.1526	0.3738	-0.6232	0.8647	0.0290
I ⇒ EGO		0.9342	0.6113	-0.3055	2.0966	0.1398
E ⇒ EGO	Total	-0.0371	0.3156	-0.6358	0.5966	-0.0076
A ⇒ EGO		-0.4310	0.4687	-1.3201	0.5142	-0.0665
C ⇒ EGO		0.0959	0.3327	-0.5173	0.7832	0.0177
ES ⇒ EGO		0.1570	0.3552	-0.5700	0.8343	0.0298
I ⇒ EGO		0.9036	0.5085	-0.0909	1.9059	0.1352

HS–hope for success, E–extraversion, A–agreeableness, C–conscientiousness, ES–emotional stability, I–intellect, EGO–ego orientation, β–standardized value

In the case of athletes’ ego orientation, none of the indirect effects obtained statistical significance (Table 6). Thus, hope for success is not a mediator in the relationship between personality traits and ego orientation of athletes.

A moderated mediation analysis was also carried out, with personality traits as the independent variables, task orientation as the mediator, and hope for success as the dependent variable (*personality traits–task orientation–hope for success*), and sport type as a moderator. None of the interaction effects (moderation) reached statistical significance (*p*>0.05). Significant indirect effects (average effects) were found for intellect (*b* = 0.335, 95% CI [0.096; 0.662], *β* = 0.034) and for conscientiousness (*b* = 0.236, 95% CI [0.046; 0.484], *β* = 0.029). No statistically significant indirect effects were found for extraversion (*b =* -0.004, 95% CI [-0.169; 0.173], *β* = -0.001), emotional stability (*b* = -0.001, 95% CI [-0.190; 0.183], *β* = 0.001), and agreeableness (*b* = 0.140, 95% CI [-0.065; 0.388], *β* = 0.015). When ego orientation was the mediator, none of the indirect effects reached statistical significance: extraversion (*b* = 0.001, 95% CI [-0.048; 0.051], *β* = 0.001), agreeableness (*b* = 0.001, 95% CI [-0.110; 0.086], *β* = 0.001), conscientiousness (*b* = 0.001, 95% CI [-0.057; 0.052], *β* = 0.001), emotional stability (*b* = 0.001, 95% CI [-0.063; 0.053], *β* = 0.001), and intellect (*b* = -0.002, 95% CI [-0.162; 0.147], *β* = 0.001). A greater number of significant indirect effects (four effects) were reported for the analysis presented earlier, where–in line with the presented assumptions–hope for success served as the mediator. Notably, when task orientation was the dependent variable and hope for success acted as a mediator, the sizes of the indirect effects were larger. However, in the case of ego orientation, whether as a dependent variable or mediator, the indirect effects did not reach statistical significance. The results of the analysis thus provide stronger empirical justification for the relationship *personality traits–hope for success–task orientation*, with hope acting as a mediator, as opposed to the relationship with task orientation as a mediator.

## Discussion

The main aim of the study was to examine the mediating role of hope for success between personality traits and goal orientation in sport. In the first place, we analyzed the correlations between the investigated variables. The positive relationships we found of extraversion, conscientiousness, emotional stability, intellect, with hope for success remain relatively consistent with the findings of other authors [26–30]. Similarly, the observed positive relationships of hope for success, conscientiousness, intellect, with task orientation are also in line with the predictions. Hence, as assumed, certain components of the constructs under investigation converge to some degree.

Hope for success emerged as a significant mediator in the relationship between conscientiousness, emotional stability, extraversion, intellect, and task orientation in sport. It was shown that higher levels of conscientiousness, emotional stability, extraversion and intellect positively correlate with hope for success, which, in turn, is positively related with task orientation in sport. The relationships found between personality traits and hope for success constitute the first path of the indirect effect in our mediation model. These traits can foster effective goal achievement, which, in turn, is conducive to the development of hope for success. For example, perseverance and achievement orientation related with conscientiousness may promote high performance in goal achievement. Similarly, higher levels of emotional resilience and less negative emotionality related to emotional stability may promote a greater ability to cope with stress, tolerate states of frustration or anger during the pursuit of difficult (challenging) goals and, as a consequence, result in more attempts and less discouragement in the face of setbacks and failures. Positive emotions, activity and sociability related to extraversion may facilitate coping with challenging goals (problems). Previous research has reported positive relationships between extraversion and favourable task-oriented coping with stress among football players [55]. It is also likely that those who are more extraverted-sociable may also receive more support from others in difficult situations. Athletes with high levels of intellect show, among other qualities, that they are more open, more likely to seek new solutions and have a more creative mindset. These traits can therefore initially help them to achieve their goals more effectively.

In turn, according to Snyder’s theory, people who are effective at achieving their goals may have a more favourable history of success, having been more likely to succeed in solving problems in the pursuit of their goals. As a result, they experience a sense of success and positive emotions more often, and more often develop positive thoughts about themselves as effective people, which, according to Snyder’s theory, reinforces the hope for success [41]. In addition, extraversion and emotional stability, associated with experiencing positive emotions, may facilitate the development of high self-esteem and positive thoughts about achieving goals. As a result of their achievement effects, these individuals are also more likely to receive positive feedback and support from their caregivers, which also may increase the chances of developing higher hope for success. In the end, when at some point in their lives they enter sport environment, they may already have an appropriate foundation (hope for success) for the development of task orientation that is beneficial in sport. The positive relationship of hope with task orientation obtained in our study (the second path of the indirect effect in our mediation model) corresponds to the correlation found by Roedel et al. [42]. Task orientation, similarly to hope for success, correlates with perseverance, orienting towards personal growth, and developing new skills [56]. What is also possible is that athletes with high hope for success, thanks to their involvement and effects, also receive more positive feedback, which is conducive to higher task orientation.

There was no mediation effect obtained for hope for success in the relationship between agreeableness and task orientation of athletes. This relationship, however, has hardly been conclusive in the past, as in fact only some of the researchers cited earlier obtained correlations of agreeableness with hope [26–28]. Besides, possibly, the impact of agreeableness on functioning in sport is lower than that of the other qualities. This point, however, remains to be further examined. Hope for success was also not observed to mediate the relationship between personality traits and ego orientation. This mostly resulted from the absence of relationship between hope for success and ego orientation. It thus appears that, even in sport, beliefs about finding solutions in demanding situations rather promote an orientation towards effort and task completion (task orientation), as opposed to a confrontational approach (ego orientation). No moderating role of the type of sport (high performance/recreational) emerged. A possible explanation for this is that the recreational athletes in our study also participated in sport activities involving competition and at times also participated in sport tournaments. The requirements of competitive sport were therefore not so foreign to them. Such issues, however, still merit more investigation.

Taken together, in our study hope for success has been shown to mediate the relationship between personality traits and task orientation conducive to effective functioning in sport. Being more genetically determined, personality traits may underlie the hope for success that evolves from early childhood. This, in turn, appears to be an important component on which, in interaction with positive influences from coaches, a task orientation beneficial to sport can be shaped in athletes. Our research also highlights the importance of personality predispositions that are relevant to sport (such as high levels of conscientiousness, extraversion, openness and emotional stability) and which provide a biological background for the development of other beneficial qualities.

Yet, the study is not free from limitations. An important limitation of the study is its cross-sectional nature. This points to the need for caution when making cause-and-effect inferences. Hence, at a further stage, the reported mediation relationships are worth verifying, for example, with longitudinal studies. In addition, in subsequent studies it would be worthwhile at least to include more homogeneous groups, which would ensure greater control of certain variables. The presence and/or selection of mediators that explain the relationship between the independent variables and the dependent variable is at times in such type of studies also subject to some doubt. In the light of the ongoing research on the relationship between personality and achievement goals/goal orientations, an attempt is often made to determine the extent to which goal orientations differ from personality traits. Despite the convergence in some of the components of these constructs, however, the correlations found between them are far from ideal [35]. Therefore, considering these issues and above all the theoretical assumptions, we believe it is reasonable to propose the positioning of the mediators between personality traits and goal orientation in sport. In addition, the study used solely self-report questionnaire techniques, which limits the objectivity of the results obtained. In another vein, other studies have also investigated the mediating role of goal orientation between personality traits and achievement [37]. By adding a construct of sport achievement that is measured in a more objective manner, further research of this nature in sport would greatly increase the chances of making the question that is being examined more objective.

Finally, although the dichotomous model of goal orientation is still prevalent, it narrows the possibility of analyzing goals to two primary factors (task-mastery, ego-performance). Therefore, it would be worthwhile to extend this research to include some of the more recent approaches to achievement goals. Subsequent approaches have extended the two-factor (dichotomous) model and included the approach/avoidance construct. The inclusion of this construct would essentially extend our analysis of the determinants of goal orientation in sport, specifically with respect to goal avoidance, which assumes that athletes can avoid e.g. doing worse than previously (mastery-avoidance) and avoid doing worse than others (performance-avoidance) [57, 58]. Notably, it would be valuable to apply the latest 3 x 2 achievement goal model, which also takes these issues into account [59]. It adopts a 6-factor approach, which further subdivides the factor mastery into task and self, where task-approach applies to effective task performance, and self-approach refers to performing better than before. Task-avoidance, on the other hand, addresses avoiding ineffective task performance, and self-avoidance refers to avoiding poorer task performance than earlier. Accordingly, in the 3x2 model, achievement goals are identified in more detail, i.e. task-approach/avoidance, self-approach/avoidance, other-approach/avoidance [59, 60]. This model has already been successfully applied in a sport context [60–63]. Applying this more fine-grained approach in our analysis would contribute to a deeper understanding of the conditionings of goal orientation in sport.

## Supporting information

S1 Checklist Checklist of items that should be included in reports of observational studies. (PDF)

S1 File Data used in analysis. (ZIP)
